# Climate impact from diet in relation to background and sociodemographic characteristics in the Västerbotten Intervention Programme

**DOI:** 10.1017/S1368980019002131

**Published:** 2019-09-30

**Authors:** Anna Strid, Elinor Hallström, Therese Hjorth, Ingegerd Johansson, Bernt Lindahl, Ulf Sonesson, Anna Winkvist, Ena Huseinovic

**Affiliations:** 1 Department of Internal Medicine and Clinical Nutrition, the Sahlgrenska Academy, University of Gothenburg, Gothenburg, Sweden; 2 Department of Agrifood and Bioscience, RISE- Research Institutes of Sweden, Gothenburg, Sweden; 3 Cariology, Department of Odontology, Umeå University, Umeå, Sweden; 4 Occupational and Environmental Medicine, Department of Public Health and Clinical Medicine, Umeå University, Umeå, Sweden

**Keywords:** Climate impact, Sociodemographic factors, Carbon dioxide equivalents, Food, Diet

## Abstract

**Objective:**

The objective of this study was to examine climate impact from diet across background and sociodemographic characteristics in a population-based cohort in northern Sweden.

**Design:**

A cross-sectional study within the Västerbotten Intervention Programme. Dietary data from a 64-item food frequency questionnaire collected during 1996–2016 were used. Energy-adjusted greenhouse gas emissions (GHGE) for all participants, expressed as kg carbon dioxide equivalents/day and 4184 kJ (1000 kcal), were estimated using data from life cycle analyses. Differences in background and sociodemographic characteristics were examined between participants with low and high GHGE from diet, respectively. The variables evaluated were age, BMI, physical activity, marital status, level of education, smoking, and residence.

**Setting:**

Västerbotten county in northern Sweden.

**Participants:**

In total, 46 893 women and 45 766 men aged 29–65 years.

**Results:**

Differences in GHGE from diet were found across the majority of examined variables. The strongest associations were found between GHGE from diet and age, BMI, education, and residence (all *P* < 0·001), with the highest GHGE from diet found among women and men who were younger, had a higher BMI, higher educational level, and lived in urban areas.

**Conclusions:**

This study is one of the first to examine climate impact from diet across background and sociodemographic characteristics. The results show that climate impact from diet is associated with age, BMI, residence and educational level amongst men and women in Västerbotten, Sweden. These results define potential target populations where public health interventions addressing a move towards more climate-friendly food choices and reduced climate impact from diet could be most effective.

The food and agricultural sector has a significant role in greenhouse gas emissions (GHGE)^(^ ^1^ ^–^ ^4^ ^)^. Food production contributes between 19 and 29% of total GHGE worldwide today^(^ ^5^ ^)^. In Sweden, the food sector accounts for about 25% of all emissions^(^ ^6^ ^)^. There are significant options to reduce GHGE from food production by reduced waste, new technology and improved management. Springmann and colleagues estimate the potential for reducing GHGE globally by such measures to be in the range 13–18%^(^ ^3^ ^)^. However, in Sweden and in most parts of Europe, productivity is already high and residual potential for reducing GHGE through increased productivity and technical solutions is not as significant^(^ ^7^ ^,^ ^8^ ^)^. For these countries, changes in dietary habits and food intake are more important as they have great potential to reduce total GHGE^(^ ^9^ ^–^ ^11^ ^)^. However, to achieve a reduced climate impact from diet, more information is needed on where, and among who, public health messages for reduced dietary carbon footprint should be directed^(^ ^12^ ^)^.

Relatively few studies have examined how climate impact from diet relates to background and sociodemographic characteristics. A study from Ireland evaluated GHGE from diet among Irish adults and found that males, younger consumers and individuals with secondary education had higher GHGE from diet compared with women, older consumers and individuals with either tertiary education or below secondary education^(^ ^13^ ^)^. Another study among 5364 Swedish participants aged 18–45 years also found lower GHGE from diet among women than men; however, in this study, GHGE from diet increased with age^(^ ^14^ ^)^. This was also found in a study from America within the National Health and Nutrition Examination Study 2005–2010, where individuals were divided into quintiles depending on energy-adjusted GHGE from diet. Adult women as well as younger individuals (18–29 years) were more likely to have diets in the lowest quintile group^(^ ^15^ ^)^. The most recent national food survey in Sweden from 2010 to 2011 found that consumption of different food groups varies between groups in the population depending on, for example, gender, age, level of education and BMI^(^ ^16^ ^)^. The results showed that women have higher intake of fruit and vegetables than do men and that men eat more meat, fish, and dairy products than do women. Furthermore, the findings showed that older men (65–80 years) generally eat less meat than younger men (18–64 years), and that individuals with a university education have a higher intake of fruit, vegetables, cheese, and alcohol compared with those with a lower level of education.

Through national food surveys in Sweden, food consumption across different groups in the population has been mapped^(^ ^16^ ^)^. However, less is known regarding differences in climate impact from diet across background and sociodemographic variables. This information is needed to better understand how and why GHGE from diet differ within the population and to identify target populations for public health interventions to reduce climate impact from diet. Hence, the aim of this paper was to identify how different background and sociodemographic characteristics relate to climate impact from diet among women and men within a population-based cohort in northern Sweden.

## Methods

### Study design and participants

The Västerbotten Intervention Programme (VIP) is an ongoing, population-based prospective study that was initiated 1985 and runs in the county of Västerbotten in northern Sweden^(^ ^17^ ^)^. The county has approximately 260 000 inhabitants, of which more than 120 000 live in the city of Umeå. Every year, all inhabitants turning 40, 50, and 60 years are invited to participate in the study. The study participants receive a mailed invitation letter inviting them to their local health centre for a standardized medical examination. They also complete a comprehensive questionnaire on diet and lifestyle. The participation rate has varied over time, with an average of 60%. Reports of significant social selection bias in relation to age, unemployment or income have been limited^(^ ^18^ ^)^. Until 1996, 30-year olds were also invited, but for financial reasons today this only persists in some communities^(^ ^17^ ^)^. The Research Ethics Committee at Umeå University approved the original study in 1984, and the Regional Ethics Examination Board in Gothenburg approved the current study in 2017. Written informed consent was obtained from all participants.

### Dietary assessment

At the health examination within VIP, participants complete a 64-item food frequency questionnaire (FFQ) including nine frequencies, ranging from never to ≥4 times per day. The FFQ is semi-quantitative and includes four pictures with increasing amounts of meat, vegetables, and potato/rice/pasta to indicate portion sizes of these three food groups. For other foods, either gender- or age-specific portion sizes or predetermined sizes, such as a fruit, are used^(^ ^17^ ^)^. Before 1996, a longer version of the FFQ was used, with questions on 84 foods. The longer version of the FFQ has been validated against ten repeated 24-h recalls among 246 study participants^(^ ^19^ ^)^. The validation study showed that the FFQ captures a relatively higher intake of dairy products, bread, cereals, vegetables, fruit, rice, potatoes, and pasta, and a relatively lower intake of meat, fish, sweets, and alcohol, than the 24-h recalls. Spearman’s correlation coefficients between the two methods ranged from 0·15 to 0·69 for different food groups, which is in line with other similar prospective cohort studies using FFQ to measure dietary intake^(^ ^19^ ^)^.

### Sample selection

For the current paper, only participants who had completed the short version of the FFQ were included, i.e. from 1996 to 2016. Participants were excluded if the food intake level was below the first percentile or over the 99th percentile calculated separately for women and men, and if data on body weight were missing. Food intake level was calculated as the estimated total energy intake divided by basal metabolic rate according to Schofield’s equation^(^ ^20^ ^)^. Furthermore, if more than 10% of the answers from the FFQ were missing, and/or if any of the three questions indicating portion size were not filled out, individuals were excluded. Finally, individuals under the age of 29 and over 65 years, and with a height of less than 130 cm or above 210 cm, a body weight less than 35 kg, or BMI below 15·0 kg/m^2^ were also excluded, in line with previous publications from the VIP study.

### Estimation of dietary climate impact

Estimations of GHGE from diet were made for all food items in the FFQ. All 64 foods were categorized into ten main food groups; fats; cereals, rice and potato; fruit and berries; vegetables; dairy products; meat; poultry; fish; sugar-containing foods and snacks, and beverages. As GHGE varied within these groups, the ten main food groups were further divided into 53 subgroups. Life cycle analysis data from scientific publications and compilations, as well as from the climate database from the Research Institutes of Sweden^(^ ^8^ ^,^ ^21^ ^–^ ^32^ ^)^, were used to estimate GHGE for all subgroups of foods, see Supplemental Table S1. To capture variations in GHGE within a subgroup, emission values were for some subgroups based on consumption weighted averages reflecting differences in emission values due to type of food (e.g. between beef, lamb, pork, and game in the subgroup ‘Meat’), and production method (e.g. between land-based production, unheated, and heated greenhouse production in the subgroup ‘Tomatoes and cucumbers’); this is further explained in the Supplementary Material.

Carbon dioxide equivalents (CO_2_e) were used to indicate GHGE of various foods, with the functional unit kg CO_2_e/kg edible food product. For foods consumed in prepared form (e.g. pasta, beans, meat), the functional unit refers to cooked weight. Emissions caused by producing food that is wasted throughout the life cycle, including consumer waste (e.g. food wasted at home or restaurant), are included in the overall estimation of the climate impact of food. Hence, the GHGE values represent the climate impact from the production of 1 kg of food reaching the consumer, including the food wasted along the food chain. The system limits used were primary production (including production of inputs such as, for example, fertilizers and fuels) up to and including the retail phase. Emissions after retail phase such as consumer transportation, storing, cooking, and waste management (e.g. collection, sorting, incineration, recycling) were not included, nor were emissions related to land-use change. If the life cycle analysis data did not include the same system boundaries, standard emissions were added for different stages in the food system^(^ ^22^ ^)^. Calculations were based on global warming potential factors from the fourth assessment report by the Intergovernmental Panel on Climate Change (IPCC) from 2007^(^ ^33^ ^)^. However, for animal-based foods and rice, updated global warming potential factors for methane from the fifth assessment report (IPCC, 2014) were used^(^ ^1^ ^)^. After the GHGE for all individual foods included in the FFQ had been estimated, the total GHGE from diet were calculated for all study participants, expressed in kg CO_2_e/day.

### Non-dietary variables

The background and sociodemographic variables examined in relation to climate impact of food were: age, BMI, physical activity, marital status, level of education, smoking, and residence. Information about these variables was collected during the standardized medical examination and through the comprehensive questionnaire on diet and lifestyle. BMI was examined both as a continuous and categorical variable and was calculated as:



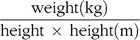




Physical activity was measured using the Cambridge index of physical activity, which is a validated index based on questions regarding physical activity during working hours and free time, respectively(34). For participants with a missing value for any of the two questions, this was replaced with the lowest intensity level for that variable. The four categories used for analysis were inactive, moderately inactive, moderately active, and active. For marital status the four categories used for analysis were unmarried, married/cohabitant, divorced/separated, and widow/widower. Smoking status was originally divided into five categories; smoker; ex-smoker; non-smoker; smokes sometimes, and smoked sometimes in the past. For the current analysis the five categories were merged into three categories; currently smoking; has smoked, and does not smoke. Furthermore, the variable residence consisted of urban and rural areas. If the study participants lived in the three most populous cities Umeå, Skellefteå or Lycksele, this was categorized as urban area, whereas smaller cities and the countryside were referred to as a rural area. For level of education three categories were used for the analysis: basic level of 9 years, high school, and university. The category ‘basic level of 9 years’ was created by merging two categories in the original variable: one reflecting current educational system and one reflecting an older educational system.

### Statistical analysis

Both energy-adjusted and unadjusted GHGE from diet were examined in relation to background and sociodemographic variables. Only energy-adjusted results are presented as similar results were found for both analyses; however, differences that did emerge are mentioned in the results section. The total climate impact of diet was energy-adjusted by using the following equation:



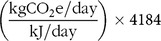




Normally distributed variables are presented using mean and sd. Non-normally distributed variables are presented using median and the first and third quartiles. Women and men were ranked and divided into quintiles based on their overall GHGE from diet. Thus, quintile one represents women and men with the lowest GHGE from diet, and quintile five represents women and men with the highest GHGE. Differences in background and sociodemographic variables between quintiles one and five were examined using Mann-Whitney U test for non-normally distributed variables, and Student t-test for normally distributed variables. For categorical variables, chi-square tests were used.

Type III tests based on the General Linear Model procedure were used to evaluate associations between background and sociodemographic variables and GHGE from diet, given all covariates in the model. The variables evaluated in the model were age, BMI, physical activity, marital status, level of education, smoking, and residence. All variables were added to the model as categorical variables except for age and BMI, which were examined as continuous variables. GHGE from diet was log transformed to correct for skewness before being entered into the model. All analyses were stratified on gender. For statistical analyses, SPSS Version 24 (IBM SPSS Statistics) was used. Statistical significance was set to *P* < 0·05.

## Results

### Study participants

In total, 49 710 women and 48 644 men were included in VIP during 1996–2016. After exclusion based on previously mentioned exclusion criteria related to food intake level, age, height, weight, and BMI, 46 893 women and 45 766 men remained. The age range for women was 29–65 years, and 29–63 years for men. Among women, 38% reported to have attained a university degree, and mean BMI was 25·8 kg/m^2^. The corresponding numbers for men were 38% and 26·8 kg/m^2^, respectively. Background information for the included participants can be found in Table 1.

**Table 1:** Background and sociodemographic characteristics of individuals participating in the Västerbotten Intervention Programme during 1996–2016 (*n* 92 659)

Variable	Women (*n* 46 893)		Men (*n* 45 766)	
	Mean, median or %	sd or 1st; 3rd quartile	Mean, median or %	sd or 1st; 3rd quartile
Age* (y)	49·9	40·1; 59·6	49·9	40·1; 59·6
Weight†,‡ (kg)	70·6	13·6	85·8	13·8
Height†, ‡ (cm)	165·2	6·1	178·9	6·7
BMI†,‡ (kg/m^2^)	25·8	4·8	26·8	3·9
Underweight, <18·5 (%)	1·1	–	0·3	–
Normal, 18·5–25·0 (%)	50·2	–	34·2	–
Overweight, 25·0–30·0 (%)	31·8	–	48·5	–
Obese, >30·0 (%)	16·9	–	17·0	–
Physical activity§ (%)				
Inactive	17·5	–	18·1	–
Moderately inactive	29·0	–	28·3	–
Moderately active	27·1	–	29·0	–
Active	26·0		24·2	
Marital status§ (%)				
Unmarried	7·5	–	13·0	–
Married/cohabitant	80·5	–	78·7	–
Divorced/separated	9·1	–	7·0	–
Widow/widower	2·2	–	0·6	–
Level of education§ (%)				
Basic level, 9 years	28·9	–	34·3	–
High school	33·0	–	38·4	–
University	37·6	–	26·8	–
Smoking§ (%)				
Currently smoking	17·8	–	16·2	–
Have smoked	30·8	–	30·5	–
Do not smoke	50·7	–	51·8	–
Residence§ (%)				
Urban area	74·3	–	73·2	–
Rural area	24·7	–	25·8	–
Energy intake†,‡ (kJ/day)	6067	1799	8075	2552
Climate impact from diet* (kg CO_2_e/day)	2·9	2·3; 3·5	3·6	2·9; 4·5

* Presented using median and first; third quartiles.

† Presented using mean and sd.

‡ Adjusted for age and year of study participation.

§ Missing values for; physical activity, *n* 352 (women *n* 206, men *n* 146); marital status, *n* 610 (women *n* 313, men *n* 297); level of education, *n* 490 (women *n* 288, men *n* 202); smoking, *n* 1013 (women *n* 349, men *n* 664); residence, *n* 931 (women *n* 471, men *n* 460).

### Background and sociodemographic variables related to energy-adjusted GHGE from diet

Importantly, as seen in Table 2 **,** men with the highest GHGE from diet, represented in quintile five, were characterized by being younger, having a higher BMI, higher educational level, and by living more often in urban compared with rural areas than men with the lowest GHGE from diet, represented in quintile one, all *P* < 0·001. For example, men in quintile five had a median age of 40·3 years compared with 50·3 years among men in quintile one. Similarly**,** women with the highest GHGE (quintile five) were younger, had higher BMI, were married/cohabitant more often, had higher educational level, and lived more often in urban compared with rural areas than women with the lowest GHGE (quintile one), all <0·001 (Table 3). As an example, women in quintile five had a mean BMI of 26·6 kg/m^2^ compared with 25·2 kg/m^2^ among women in quintile one.

**Table 2:** Background and sociodemographic characteristics for men in quintiles one and five based on energy-adjusted greenhouse gas emissions from diet within the Västerbotten Intervention Programme (*n* 18 306)

Variable	Quintile one (*n* 9153)		Quintile five (*n* 9152)		
	Mean, median or %	sd or 1st; 3rd quartile	Mean, median or %	sd or 1st; 3rd quartile	*P*-value
Age* (y)	50·3	40·7; 60·0	40·3	40·0; 50·1	<0·001§
Weight† (kg)	83·0	12·9	89·1	14·8	<0·001∥
BMI† (kg/m^2^)	26·2	3·7	27·7	4·2	<0·001∥
BMI, subgroups (kg/m^2^)					<0·001¶
Underweight, <18·5 (%)	0·4	–	0·3	–	
Normal, 18·5–25·0 (%)	39·9	–	26·0	–	
Overweight, 25·0–30·0 (%)	47·0	–	50·1	–	
Obese, >30·0 (%)	12·8	–	23·6	–	
Physical activity‡ (%)					<0·001¶
Inactive	18·6	–	18·6	–	
Moderately inactive	29·3	–	26·4	–	
Moderately active	29·2	–	28·8	–	
Active	22·4	–	25·9	–	
Marital status‡ (%)					<0·001¶
Unmarried	14·6	–	14·7	–	
Married/cohabitant	77·2	–	76·1	–	
Divorced/separated	6·7	–	8·0	–	
Widow/widower	0·8	–	0·5	–	
Level of education‡ (%)					<0·001¶
Basic level, 9 years	47·3	–	24·4	–	
High school	28·4	–	45·7	–	
University	23·7	–	29·5	–	
Smoking‡ (%)					<0·001¶
Currently smoking	16·0	–	17·8	–	
Have smoked	33·8	–	27·3	–	
Do not smoke	48·8	–	53·5	–	
Residence‡ (%)					<0·001¶
Urban area	69·8	–	76·6	–	
Rural area	29·5	–	22·1	–	
Energy intake† (kJ/day)	8786	2636	7531	2594	<0·001∥
Climate impact from diet/4184 kJ* (kg CO_2_e/day and 4184 kJ)	1·5	1·4; 1·6	2·5	2·4; 2·7	<0·001§

CO_2_e, carbon dioxide equivalents.

* Presented using median and first; third quartiles.

† Presented using mean and sd.

‡ Missing values for; physical activity quintile one *n* 42, quintile five *n* 27; marital status quintile one *n* 64, quintile five *n* 60; level of education quintile one *n* 50, quintile five *n* 32; smoking quintile one *n* 125, quintile five *n* 135; residence quintile one *n* 68, quintile five *n* 120.

§ Estimated using Mann–Whitney U test.

∥ Estimated using a Student *t* test.

¶ Estimated using chi-square test.

**Table 3:** Background and sociodemographic characteristics for women in quintiles one and five based on energy-adjusted greenhouse gas emissions from diet within the Västerbotten Intervention Programme (*n* 18 759)

Variable	Quintile one (*n* 9380)		Quintile five (*n* 9376)		
	Mean, median or %	sd or 1st; 3rd quartile	Mean, median or %	sd or 1st; 3rd quartile	*P*-value
Age* (y)	50·0	40·1; 59·6	40·6	40·0; 50·4	<0·001§
Weight† (kg)	68·5	13·1	73·0	14·2	<0·001∥
BMI† (kg/m^2^)	25·2	4·6	26·6	5·0	<0·001∥
BMI, subgroups (kg/m^2^)					<0·001¶
Underweight, <18·5 (%)	1·6	–	0·7	–	
Normal, 18·5–25·0 (%)	55·5	–	43·4	–	
Overweight, 25·0–30·0 (%)	29·2	–	34·0	–	
Obese, >30·0 (%)	13·6	–	21·9	–	
Physical activity‡ (%)					<0·001¶
Inactive	16·7	–	18·4	–	
Moderately inactive	31·5	–	26·7	–	
Moderately active	27·1	–	25·6	–	
Active	24·2	–	28·9	–	
Marital status‡ (%)					<0·001¶
Unmarried	9·2	–	7·3	–	
Married/cohabitant	77·6	–	81·1	–	
Divorced/separated	10·1	–	9·1	–	
Widow/widower	2·5	–	1·9	–	
Level of education‡ (%)					<0·001¶
Basic level, 9 years	33·0	–	21·7	–	
High school	30·1	–	37·2	–	
University	36·1	–	40·5	–	
Smoking‡ (%)					<0·001¶
Currently smoking	16·8	–	18·4	–	
Have smoked	29·9	–	33·3	–	
Do not smoke	52·5	–	47·4	–	
Residence‡ (%)					<0·001¶
Urban area	71·0	–	77·5	–	
Rural area	28·0	–	21·4	–	
Energy intake† (kJ/day)	6485	1883	5648	1757	<0·001∥
Climate impact from diet/4184 kJ* (kg CO_2_e/day and 4184 kJ)	1·6	1·5; 1·7	2·6	2·5; 2·8	<0·001§

CO_2_e, carbon dioxide equivalents.

* Presented using median and first; third quartiles.

† Presented using mean and sd.

‡ Missing values for; physical activity quintile one *n* 49, quintile five *n* 34; marital status quintile one *n* 60, quintile five *n* 57; level of education quintile one *n* 68, quintile five *n* 60; smoking quintile one *n* 68, quintile five *n* 83; residence quintile one *n* 94, quintile five *n* 106.

§ Estimated using Mann–Whitney U test.

∥ Estimated using Student *t* test.

¶ Estimated using a chi-square.

### Association among individual characteristics and energy-adjusted GHGE from diet

Among men, age (*P* < 0·001), BMI (*P* < 0·001), physical activity (*P* = 0·018), marital status (*P* < 0·001), educational level (*P* < 0·001), smoking (*P* < 0·001), and residence (*P* < 0·001) were all associated with GHGE from diet (Table 4), and together explained 10·0% of the variation in energy-adjusted GHGE from diet. Among these variables, age, BMI, and level of education explained the most variation in GHGE from diet (range 0.3–5.7%, Table 4). Among women, age (*P* < 0·001), BMI (*P* < 0·001), physical activity (*P* < 0·001), marital status (*P* = 0·008), educational level (*P* < 0·001), smoking (*P* < 0·001), and residence (*P* < 0·001) were all associated with GHGE from diet (see Table 5), and together explained 2.6% of the variation in energy-adjusted GHGE from diet. Among these, BMI, age, level of education, and residence explained the most variation in GHGE from diet (range 0·3–1·5%, Table 5).

**Table 4:** Association between energy-adjusted climate impact from diet and background and sociodemographic characteristics for men within the Västerbotten Intervention Programme (*n* 45 766)*

Variable	*n*	Median (kg CO_2_e/day/4184 kJ)	1st; 3rd quartile (kg CO_2_e/day/4184 kJ)	*R*^2^† (%)	*P*-value
Age (y)				5·9	<0·001
29–34	652	1·98	1·78; 2·22		
35–44	19 025	2·05	1·82; 2·32		
45–54	14 063	1·94	1·73; 2·20		
55–65	12 026	1·79	1·58; 2·03		
BMI, subgroups (kg/m^2^)				2·3	<0·001
Underweight, <18·5	121	1·85	1·58; 2·17		
Normal, 18·5–25·0	15 740	1·90	1·68; 2·15		
Overweight, 25·0–30·0	22 112	1·96	1·73; 2·23		
Obese, >30·0	7793	2·03	1·79; 2·33		
Physical activity				0·0	0·018
Inactive	8303	1·95	1·71; 2·23		
Moderately inactive	12 964	1·93	1·71; 2·19		
Moderately active	13 295	1·95	1·72; 2·22		
Active	11 058	1·97	1·74; 2·24		
Missing value	146	1·87	1·61; 2·14		
Marital status				0·1	<0·001
Unmarried	5968	1·94	1·70; 2·25		
Married/cohabitant	36 009	1·95	1·72; 2·21		
Divorced/separated	3211	1·99	1·74; 2·27		
Widow/widower	281	1·88	1·65; 2·16		
Missing value	297	1·94	1·72; 2·26		
Level of education				0·4	<0·001
Basic level, 9 years	15 709	1·86	1·64; 2·12		
High school	17 567	2·00	1·78; 2·28		
University	12 288	1·98	1·75; 2·25		
Missing value	202	1·90	1·67; 2·15		
Smoking				0·2	<0·001
Currently smoking	7424	1·96	1·72; 2·25		
Have smoked	13 961	1·92	1·70; 2·19		
Do not smoke	23 717	1·96	1·73; 2·23		
Missing value	664	1·95	1·72; 2·23		
Residence				0·3	<0·001
Urban area	33 513	1·96	1·73; 2·23		
Rural area	11 793	1·91	1·69; 2·17		
Missing value	460	2·01	1·77; 2·31		

CO_2_e, carbon dioxide equivalents.

* Type III tests, based on the General Linear Model procedure, were used to evaluate associations between *a priori* selected variables and GHGE from diet, given all covariates in the model. GHGE from diet were log transformed to correct for skewness. Study participants who did not have complete information for all covariates were also used in the model.

† Adjusted.

**Table 5:** Association between energy-adjusted greenhouse gas emissions from diet and background and sociodemographic characteristics for women within the Västerbotten Intervention Programme (*n* 46 893)*

Variable	*n*	Median (kg CO_2_e/day/4184 kJ)	1st; 3rd quartile (kg CO_2_e/day/4184 kJ)	*R*^2^† (%)	*P*-value
Age (y)				0·3	<0·001
29–34	635	1·92	1·70; 2·15		
35–44	19 702	2·09	1·87; 2·36		
45–54	14 296	1·99	1·78; 2·23		
55–65	12 260	2·03	1·82; 2·26		
BMI, subgroups (kg/m^2^)				1·5	<0·001
Underweight, <18·5	501	1·95	1·72; 2·20		
Normal, 18·5–25·0	23 540	2·02	1·80; 2·25		
Overweight, 25·0–30·0	14 917	2·06	1·84; 2·32		
Obese, >30·0	7935	2·10	1·87; 2·38		
Physical activity				0·1	<0·001
Inactive	8193	2·06	1·83; 2·31		
Moderately inactive	13 591	2·02	1·80; 2·27		
Moderately active	12 703	2·04	1·82; 2·28		
Active	12 200	2·06	1·84; 2·32		
Missing value	206	1·97	1·79; 2·25		
Marital status				0·1	0·008
Unmarried	3502	2·01	1·78; 2·28		
Married/cohabitant	37 762	2·05	1·83; 2·30		
Divorced/separated	4275	2·03	1·80; 2·29		
Widow/widower	1041	2·02	1·80; 2·25		
Missing value	313	2·03	1·82; 2·27		
Level of education				0·3	<0·001
Basic level, 9 years	13 533	2·00	1·79; 2·22		
High school	15 453	2·07	1·85; 2·33		
University	17 619	2·05	1·83; 2·32		
Missing value	288	2·03	1·78; 2·31		
Smoking				0·2	<0·001
Currently smoking	8343	2·05	1·83; 2·31		
Have smoked	14 438	2·06	1·83; 2·32		
Do not smoke	23 763	2·03	1·82; 2·28		
Missing value	349	2·06	1·82; 2·33		
Residence				0·3	<0·001
Urban area	34 860	2·05	1·83; 2·31		
Rural area	11 562	2·01	1·79; 2·25		
Missing value	471	2·05	1·83; 2·33		

CO_2_e, carbon dioxide equivalents.

* Type III tests, based on the General Linear Model procedure, were used to evaluate associations between *a priori* selected variables and GHGE from diet, given all covariates in the model. GHGE from diet was log transformed to correct for skewness. Study participants who did not have complete information for all covariates were also used in the model.

† Adjusted.

### Unadjusted GHGE from diet

In general, similar results were found for both energy-adjusted and unadjusted GHGE, except for BMI, residence, and physical activity. For BMI and residence, differences between individuals in quintiles one and five faded when unadjusted GHGE from diet were used compared with energy-adjusted value, such that the statistical significance for residence disappeared. For physical activity, the differences between quintiles became more distinguished, with quintile five having a higher physical activity level than quintile one when unadjusted GHGE from diet were used compared with energy-adjusted GHGE (data not shown).

## Discussion

The aim of this study was to examine GHGE from diet in relation to background and sociodemographic characteristics in a population-based sample of women and men in northern Sweden. Overall, the results demonstrate distinct differences in age, BMI, level of education, and residence across participants with high *v*. low GHGE from diet. For both genders, participants with high GHGE from diet were characterized by being younger, having a higher BMI, higher educational level, and more often living in urban areas than those with low GHGE.

The current study revealed a clear association between age and GHGE from diet, with participants aged 35–44 years having the highest, and participants aged 55–65 years the lowest, GHGE from diet. In contrast to our findings, another Swedish study recently reported GHGE from diet to increase with age^(^ ^14^ ^)^. However, that study examined a younger population within a more narrow age range (18–45 years) compared with VIP (29–65 years), and this restrains a direct comparison of results. In line with our results, a study from Ireland among 1500 individuals aged 18–87 years found that the youngest participants (aged 18–35 years) to have the highest GHGE from diet, followed by participants aged 36–50 years^(^ ^13^ ^)^. However, the authors from the Irish study stressed that differences in GHGE between sociodemographic groups were mainly affected by differences in quantity of food consumed, i.e. energy intake. Also, the Irish study found that individuals with secondary education had the highest GHGE from diet compared with individuals with either tertiary education or below secondary education^(^ ^13^ ^)^. Again, the authors highlighted that this finding was mainly influenced by differences in quantity of food consumed. In the current study, energy-adjusted GHGE from diet were therefore used in an attempt to minimize differences in GHGE related to differences in energy intake. Hence, a strict comparison of results between the two studies is not possible. Still, both studies report results in the same direction, indicating that differences in energy intake do not explain all of the variation observed. Instead, true underlying differences in food intake patterns are likely to explain some of the variation in GHGE from diet across age groups and education level in this population. The finding that individuals with high GHGE from diet also have higher educational level could possibly be explained by higher income, enabling higher and more frequent consumption of foods that are more expensive and usually generate higher GHGE such as meat, fish, and cheese. In support of this argument, the latest national food survey in Sweden found that individuals with higher income (equal to or above the median income) consume more animal products than do individuals with lower income. The survey also showed that individuals with lower income consume more sweets and snacks, i.e. foods with low GHGE^(^ ^16^ ^)^. Furthermore, individuals with either high school or university education were found to have the highest intake of cheese compared with those with lower educational level^(^ ^16^ ^)^, which may indicate generally higher GHGE from diet as cheese is one of the foods with the highest GHGE per kg food product^(^ ^25^ ^)^.

Furthermore, we found a clear positive association between GHGE from diet and BMI. As energy-adjusted GHGE were used, this finding cannot solely be explained by greater energy intake among those with higher BMI. Thus, this could indicate more climate-friendly food choices among individuals with lower BMI compared with those with higher BMI. Another explanation could be that individuals with higher BMI under-report to a higher degree^(^ ^35^ ^,^ ^36^ ^)^. As under-reporting of food intake usually involves specific foods considered ‘unhealthy’, such as sweets, cakes, pastries etc. (foods associated with low GHGE)^(^ ^37^ ^)^, this could mean that the meat and dairy share of the diet is overestimated, which would give falsely higher GHGE from diet. However, when under-reporting was examined in VIP, under-reporting men were found to have significantly fewer intakes of meat and under-reporting women of dairy products, in addition to sweet products, breads and ‘high-fat’ products^(^ ^36^ ^)^, which disputes this hypothesis. Still, the finding that the correlation between BMI and GHGE from diet was weaker in unadjusted models could be explained by a higher degree of under-reporting amongst individuals with a higher BMI^(^ ^35^ ^,^ ^36^ ^)^. Moreover, we found a stronger association between GHGE from diet and BMI among men compared with among women, which is in line with similar findings from the latest national food survey in Sweden^(^ ^16^ ^)^. The results from the current paper also show that individuals who live in urban areas have higher GHGE from diet, and consequently less climate-friendly food choices, than have individuals who live in more rural areas. This difference could potentially be due to that people living in rural areas often having less access to food stores compared with people living in urban areas. They might therefore do their grocery shopping more seldom, and consequently buy fewer goods with short durability, such as meat and dairy, which have high GHGE per kg food product^(^ ^25^ ^)^. The difference could also be because individuals in rural areas have greater access to local foods and nature and therefore hunt, fish and pick foods from the forest to a higher degree compared with individuals living in urban areas of Skellefteå and Umeå. However, as it is not possible to determine the origin of meat, fish, berries etc. from the present FFQ, our paper likely underestimates the true difference in climate impact from diet found between urban and rural residents.

In this report, we mainly present energy-adjusted GHGE, although differences that emerged when unadjusted values were used are highlighted. With unadjusted GHGE, greater emphasis is placed on quantity of foods, allowing differences in GHGE to be explained by differences in total energy intake. In contrast, energy-adjusted GHGE put greater weight on quality of foods, i.e. specific food choices. Both aspects are of importance and relevant in order to reduce climate impact of diet. However, it can be argued that food choices are easier to influence through public health campaigns rather than the amount of energy intake consumed, which often correlates with non-modifiable individual characteristics such as age and gender. By examining energy-adjusted emissions, differences in GHGE from diet that remain independent of energy intake are more clearly displayed and therefore highlighted in the current paper.

A major strength of this study is the large and population-based sample, which gives more robust results, as well as the coherent and standardized methodology used to assess food intake since 1996, which decreases the risk of measurement errors. Furthermore, the longer version of the FFQ has been validated against ten repeated 24-h recalls. However, this study also has some limitations. First, as the study is located in northern Sweden, this may affect the external validity. Second, the study population mainly consisted of individuals aged 40, 50, or 60 years; hence, the results cannot be projected to younger or older age groups. Third, the 64-item FFQ does not capture the participants’ dietary habits in their entirety because of the limited number of items included. Also, to maintain consistency over the years, the FFQ has not been updated with new foods during the time of data collection (1996–2016). Consequently, it is likely missing more foods consumed during the later years of data collection compared with the earlier years. Similarly, life cycle assessment data age quickly^(^ ^25^ ^)^ and as the data used in the current study are based on today’s food production systems, they might be more representative of the later years of data collection than the earlier. Fourth, the FFQ was not designed to capture differences in GHGE across different foods (e.g. type of meat animals, or if the fruit was grown domestically, imported, or imported by air), and might therefore combine foods with various GHGE as one food group. Correcting attempts were made by making assumptions about consumption based on national consumption statistics. However, we are aware that the correcting attempts are crude as the national consumption statistics used for all VIP intake data between 1996–2016 are from 2016 and also do not differentiate intake by for example age group. Fifth, the emission values used for GHGE for subgroups of foods to a large extent reflect the average GHGE caused by products from different sources and produced by varying production methods; hence with different amounts of CO_2_e/kg food product. Consequently, the true variation in daily diet-related GHGE might be large between individuals with similar reported diet (for example depending on the proportion of meat consumption consisting of ruminant meat). This is not critical for the results presented, but needs to be kept in mind in the broader discussion on sustainable food systems and the role of production systems and diets. Sixth, the large sample size might have increased the number of statistical significant findings. Finally, for future cohort studies to be able to examine not only the health impact of diet, but also the climate impact, dietary assessment methods need to be designed specifically for that purpose. In depth questions about foods must be included, e.g. type of meat, fish, and fruit/vegetables. Questions on type of production systems (e.g. organic/conventional or wild-caught/farmed fish) and origin (domestic, imported, or airborne-imported products) also need to be included.

## Conclusion

This study is one of the first to examine climate impact from diet across background and sociodemographic characteristics. The results show that climate impact from diet is associated with age, BMI, residence and educational level amongst men and women in Västerbotten, Sweden. These results define potential target populations where public health interventions addressing a move towards more climate-friendly food choices and reduced climate impact from diet could be most effective.

## Supplementary material

For supplementary material accompanying this paper visit https://doi.org/10.1017/S1368980019002131. click here to view supplementary material

## References

[1] Intergovernmental Panel on Climate Change (2014) Synthesis Report. Contribution of Working Groups I, II and III to the Fifth Assessment Report of the Intergovernmental Panel on Climate Change p. 151 [Core Writing Team, RK Pachauri & LA Meyer , editors]. Geneva, Switzerland: IPCC.

[2] Sonesson U , Davis J & Ziegler F (2009) Food Production and Emissions of Greenhouse Gases – An Overview of the Climate Impact of Different Product Groups. Gothenburg, Sweden: SIK, The Swedish Institute for Food and Biotechnology.

[3] Springmann M , Clark M , Mason-D’Croz D et al. (2018) Options for keeping the food system within environmental limits. Nature 562, 519–525. 30305731 10.1038/s41586-018-0594-0

[4] Hallström E , Carlsson-Kanyama A & Börjesson P (2015) Environmental impact of dietary change: a systematic review. J Cleaner Prod 91, 1–11.

[5] Vermeulen SJ , Campbell BM & Ingram JSI (2012) Climate change and food systems. Annu Rev 37, 195–222.

[6] The Swedish National Food Administration (2017) Food and Environment [Internet]. [Updated 3 October 2017; cited 27 January 2018]. https://www.livsmedelsverket.se/en/food-habits-health-and-environment/food-and-environment.

[7] Grassini P , Eskridge KM & Cassman KG (2013) Distinguishing between yield advances and yield plateaus in historical crop production trends. Nat Commun 4, 2918. 24346131 10.1038/ncomms3918 PMC3905725

[8] Bryngelsson D , Wirsenius S , Hedenus F et al. (2016) How can EU climate targets be met? A combined analysis of technological and demand-side changes in food and agriculture. Food Policy 59, 152–164.

[9] Perignon M , Masset G , Ferrari G et al. (2016) How low can dietary greenhouse gas emissions be reduced without impairing nutritional adequacy, affordability and acceptability of the diet? A modelling study to guide sustainable food choices. Public Health Nutr 19, 2662–2674. 27049598 10.1017/S1368980016000653 PMC10448381

[10] Hallström E , Röös E & Börjesson P (2014) Sustainable meat consumption: a quantitative analysis of nutritional intake, greenhouse gas emissions and land use from a Swedish perspective. Food Policy 47, 81–90.

[11] Horgan GW , Perrin A , Whybrow S et al. (2016) Achieving dietary recommendations and reducing greenhouse gas emissions: modelling diets to minimise the change from current intakes. Int J Behav Nutr Phys Act 13, 46. 27056829 10.1186/s12966-016-0370-1 PMC4823893

[12] Garnett T (2010) Where are the best opportunities for reducing greenhouse gas emissions in the food system (including the food chain)? Food Policy 37, 463–466.

[13] Hyland JJ , Henchion M , McCarthy M et al. (2017) The climatic impact of food consumption in a representative sample of Irish adults and implications for food and nutrition policy. Public Health Nutr 20, 726–738. 27667716 10.1017/S1368980016002573 PMC10261633

[14] Balter K , Sjors C , Sjolander A et al. (2017) Is a diet low in greenhouse gas emissions a nutritious diet? - analyses of self-selected diets in the LifeGene study. Arch Public Health = Archives belges de sante publique 75, 17. 28400959 10.1186/s13690-017-0185-9 PMC5385588

[15] Rose D , Heller MC , Willits-Smith AM et al. (2019) Carbon footprint of self-selected US diets: nutritional, demographic, and behavioral correlates. Am J Clin Nutr 109, 526–534. 30698631 10.1093/ajcn/nqy327 PMC6408204

[16] The Swedish National Food Administration (2012) Riksmaten 2010-11 – Livsmedels- och näringsintag bland vuxna i Sverige (Food and nutrition intake among adults in Sweden) (In Swedish). Uppsala, Sweden: Livsmedelsverket.

[17] Norberg M , Wall S , Boman K et al. (2010) The Vasterbotten Intervention Programme: background, design and implications. Global Health Action 3, 4643. 10.3402/gha.v3i0.4643 PMC2844807 20339479

[18] Weinehall L , Hallgren CG , Westman G et al. (1997) Reduction of selection bias in primary prevention of cardiovascular disease through involvement of primary health care. Scand J Prim Health Care 16, 171–176. 10.1080/028134398750003133 9800231

[19] Johansson I , Hallmans G , Wikman A et al. (2002) Validation and calibration of food-frequency questionnaire measurements in the Northern Sweden Health and Disease cohort. Public Health Nutr 5, 487–496. 12003662 10.1079/phn2001315

[20] Schofield WN (1985) Predicting basal metabolic rate, new standards and review of previous work. Human Nutr Clin Nutr 39, 5–41. 4044297

[21] Research Institutes of Sweden (2019) RISE [Internet]. Gothenburg: RISE; [updated 14 March 2019; cited 14 March 2019]. https://www.ri.se/sv.

[22] Sjörs C (2017) Näringsintag och utsläpp av växthusgaser från svenska matvanor ur ett epidemiologiskt perspektiv (Nutritional intake and greenhouse gas emissions from Swedish eating habits from an epidemiological perspective) [in Swedish] [licentiate thesis]. Stockholm, Sweden: E-print AB Stockholm. [Cited 2 February 2018].

[23] Flysjö A (2012) Greenhouse Gas Emissions in Milk and Dairy Product Chains: Improving the Carbon Footprint of Dairy Products [doctoral thesis]. Denmark: Aarhus University.

[24] Florén B , Amani P & Davis J (2017) Climate database facilitating climate smart meal planning for the public sector in Sweden. Int J Food System Dynamics 8, 72–80.

[25] Röös E (2014) Mat-klimat-listan (Food climate list) [in Swedish]. Version 1.1. Report 077. Uppsala, Sweden: SLU.

[26] Cejie J (2008) Klimatpåverkan från vilt kött (Climate Impact from Game Meat) [in Swedish] [Internet]. [Updated 5 November 2008; cited 2 March 2018]. http://ekotank.blogspot.se/2008/11/klimatpverkan-frn-vilt-ktt.html.

[27] Ziegler F , Winther U , Hognes E et al. (2013) The carbon footprint of Norwegian seafood products on the global seafood market. J Ind Ecol 17, 103–116.

[28] Winther U , Ziegler F , Hognes E et al. (2009) Carbon footprint and energy use of Norwegian seafood products. Report SFH80 A. 96068. Trondheim, Norway: SINTEF Fisheries and Aquaculture.

[29] Nilsson K , Sund V & Florén B (2011) The Environmental Impact of the Consumption of Sweets, Crisps and Soft Drinks, p. 509. Copenhagen, Denmark: Nordic Council of Ministers.

[30] Nilsson K (2010) Klimatpåverkan från bryggkaffe och snabbkaffe (Climate Impact of Filter Coffee and Instant Coffee) [in Swedish]. Report UPX00221. Gothenburg, Sweden: SIK – The Swedish Institute for Food and Biotechnology.

[31] Scarborough P , Appleby PN , Mizdrak A et al. (2014) Dietary greenhouse gas emissions of meat-eaters, fish-eaters, vegetarians and vegans in the UK. Clim Change 125, 179–192. 25834298 10.1007/s10584-014-1169-1 PMC4372775

[32] Hallström E , Håkansson N , Åkesson A et al. (2018) Climate impact of alcohol consumption in Sweden. J Clean Prod 201, 287–294.

[33] Intergovernmental Panel on Climate Change (2007) Climate Change 2007: Synthesis Report. Contribution of Working Groups I, II and III to the Fourth Assessment Report of the Intergovernmental Panel on Climate Change [Core Writing Team, RK Pachauri & A Reisinger , editors], p 104. Geneva, Switzerland: IPCC.

[34] Peters T , Brage S , Westgate K et al. (2012) Validity of a short questionnaire to assess physical activity in 10 European countries. Eur J Epidemiol 27, 15–25. 22089423 10.1007/s10654-011-9625-y PMC3292724

[35] Heitmann B & Lissner L (1995) Dietary underreporting by obese individuals – is it specific or unspecific? BMJ 311, 986–989. 7580640 10.1136/bmj.311.7011.986 PMC2550989

[36] Johansson G , Wikman Å , Åhrén AM et al. (2001) Underreporting of energy intake in repeated 24-hour recalls related to gender, age weight status, day of interview, educational level, reported food intake, smoking habits and area of living. Public Health Nutr 4, 919–927. 11527517 10.1079/phn2001124

[37] Lafay L , Mennen L , Basdevant A et al. (2000) Does energy intake underreporting involve all kinds of food or only specific food items? Results from the Fleurbaix Laventie Ville Santé (FLVS) study. Int J Obes Relat Metab Disord 11, 1500–1506. 10.1038/sj.ijo.0801392 11126348

