# Supplementary material for: Climate impact from diet in relation to background and sociodemographic characteristics in the Västerbotten Intervention Programme
Source: Public Health Nutr. 2019 Sep 30;22(17):3288–97. doi: 10.1017/S1368980019002131 (PMC10260555; doi:10.1017/S1368980019002131)
Supplement: Supplementary file 1 [file S1368980019002131sup001.docx]

**Supplementary Table 1. Greenhouse gas emissions expressed as kg carbon dioxide equivalents (CO_2_e) per kg edible food products.**

To calculate greenhouse gas emissions (GHGE) for diet, each food product was linked to data on emissions from life cycle assessment (LCA) studies. The climate impact from the LCA studies is expressed as carbon dioxide equivalents (CO_2_e) as different greenhouse gases have different global warming potential (GWP). The GWP factors are used to create the common unit CO_2_e per kg food product and are based on a 100-year time horizon. For plant-based foods, except rice, GWP factors from the 4^th^ assessment report by the Intergovernmental Panel on Climate Change (IPCC) ^(S1)^, were used in this paper, i.e., 1 for carbon dioxide, 25 for methane, and 296 for nitrous oxide. However, for animal-based foods and rice the updated GWP factor for methane, 34, from the 5^th^ assessment report (IPCC) ^(S2)^ was used.

The system boundaries used in the present paper were primary production, i.e., including emissions from agriculture (including production of inputs as, e.g., fertilizers), up to and including retail phase. Emissions after retail phase such as consumer transportation, storing, cooking, and waste management, were hence not included, nor were emissions related to land-use change. A majority of the original LCA studies that were used reported GHGE according to the selected system boundaries. In cases where original LCA studies did not use the same system boundaries, emissions were harmonized by adding standard emissions ^(S3)^ for the missing stages in the food system, e.g. transportation (between farm and retail) and packaging. In the present analyses, GHGE are calculated per kg edible food product, i.e., meat without bone, and emission values refer to food in prepared form. In cases where LCA studies presented the emission value of a raw food, re-calculations to adjust for cooking were made. Weight changes due to both hydration, i.e., cooking of rice, and dehydration, i.e., cooking of meat, were considered ^(S3-S7)^. For all foods, emissions from unavoidable food waste (i.e. shell and bone), as well as avoidable food waste along the food chain, were included. In cases were LCA studies did not include waste, adjustments were made ^(S3)^.

To capture variations in GHGE within a sub-group, emission values were based on weighted averages for some food groups. The weighted averages were used to reflect differences in emission values due to type of food within the sub groups “meat”, “fat fish”, “smoked fish”, “sausage”, “cream, crème fraiche and sour cream”, “fruits”, “berries” and “tomato and cucumber” (e.g. between beef, lamb, pork, and game in the sub-group “meat” and between different % of fat in the sub group “cream, crème fraiche and sour cream”), and to distinguish between production methods and origin in the sub groups “meat”, ”tomatoes and cucumbers”, “fruits” and “berries” (e.g., between land-based production, unheated, and heated greenhouse production in the subgroup “Tomatoes and cucumbers”). The weighted average emission was based on national consumption shares ^(S8-S10)^ and other sources ^(S4, S11)^ reflecting the average Swedish consumption. For composite dishes in the FFQ, all recipes used to define ingredients of the dish came from the national food composition database ^(S4)^ from the Swedish National Food Administration. The GHGE for the dish was estimated by either the proportion of the ingredients with the greatest importance to the weight of the dish, or the climate impact. Examples of calculation of weighted average emission based on Swedish consumption statistics, and weighted average emission of a composite dish according to a recipe from the national food composition database, can be found below the references for Supplementary Table 1.

| **Food groups** | **Food subgroups** | | **Kg CO_2_e/kg food** | **Estimated from** |
| --- | --- | --- | --- | --- |
| Fats | Butter blends | | 8.3 | Sjörs ^(S3)^, Bryngelsson et al. ^(S12)^, Flysjö ^(S13)^ |
|  | Butter | | 13 | Sjörs ^(S3)^, Bryngelsson et al. ^(S12)^, Flysjö ^(S13)^ |
|  | Vegetable oils and margarine | | 2.3 | Sjörs ^(S3)^, Bryngelsson et al. ^(S12)^ |
| Cereals, rice and potato | Bread and crisp bread | | 0.7 | Sjörs ^(S3)^, Bryngelsson et al. ^(S12)^ |
|  | Porridge | | 0.4 | RISE ^(S14)^ |
|  | Muesli and cereals | | 0.7 | Sjörs ^(S3)^, Bryngelsson et al. ^(S12)^ |
|  | Pancakes, waffles | | 1.5 | Sjörs ^(S3)^, RISE ^(S14)^ |
|  | Pizza | | 4.3 | Sjörs ^(S3)^, RISE ^(S14)^ |
|  | Potato | | 0.3 | Sjörs ^(S3)^, Bryngelsson et al. ^(S12)^ |
|  | Pasta | | 0.3 | Sjörs ^(S3)^, Bryngelsson et al. ^(S12)^ |
|  | Rice | | 0.7 | Sjörs ^(S3)^, Bryngelsson et al. ^(S12)^ |
| Fruit and berries | Berries^*^ | | 1.0 | Sjörs ^(S3)^, RISE ^(S14)^ |
|  | Fruit^†^ | | 1.4 | Sjörs ^(S3)^, Bryngelsson et al. ^(S12)^, Röös ^(S15)^ |
|  | Banana | | 2.6 | Sjörs ^(S3)^, Bryngelsson et al. ^(S12)^ |
| Vegetables | Root vegetables | | 0.2 | Sjörs ^(S3)^, Bryngelsson et al. ^(S12)^ |
|  | Tomato and cucumber^‡^ | | 1.2 | RISE ^(S14)^ |
|  | Salad, spinach, kale, broccoli | | 0.7 | Sjörs ^(S3)^, Bryngelsson et al. ^(S12)^ |
|  | Beans, pea soup | | 0.9 | Sjörs ^(S3)^, Bryngelsson et al. ^(S12)^ |
| Dairy products | Cream, crème fraiche, sour cream^§^ | | 5.7 | Sjörs ^(S3)^, Bryngelsson et al. ^(S12)^, Flysjö ^(S13)^ |
|  | White cheese 28% | | 11.8 | Sjörs ^(S3)^, Bryngelsson et al. ^(S12)^, Flysjö ^(S13)^ |
|  | White cheese 10-17% | | 10.8 | Sjörs ^(S3)^, Bryngelsson et al. ^(S12)^, Flysjö ^(S13)^ |
|  | Sour milk, yoghurt 3% | | 1.6 | Sjörs ^(S3)^, Bryngelsson et al. ^(S12)^, Flysjö ^(S13)^ |
|  | Sour milk, yoghurt 0,5% | | 1.4 | Sjörs ^(S3)^, Bryngelsson et al. ^(S12)^, Flysjö ^(S13)^ |
|  | Skimmed milk 0,5% | | 1.2 | Sjörs  ^(S3)^, Bryngelsson et al. ^(S12)^, Flysjö ^(S13)^ |
|  | Semi-skimmed 1,5% | | 1.3 | Sjörs ^(S3)^, Bryngelsson et al. ^(S12)^, Flysjö ^(S13)^ |
|  | Whole milk 3% | | 1.5 | Sjörs ^(S3)^, Bryngelsson et al. ^(S12)^, Flysjö ^(S13)^ |
|  | Ice cream | | 2.6 | RISE ^(S14)^ |
| Meat | Sausage/liverwurst^¶^ | | 15 | Sjörs ^(S3)^, Bryngelsson et al. ^(S12)^ |
|  | Meat as sandwich topping | | 9.6 | Sjörs ^(S3)^, Bryngelsson et al. ^(S12)^ |
|  | Minced meat dishes | | 18.6 | Sjörs ^(S3)^, Bryngelsson et al. ^(S12)^, RISE ^(S14)^ |
|  | Meat dish | | 6.8 | Sjörs ^(S3)^, Bryngelsson et al. ^(S12)^, Ceije ^(S16)^ |
|  | Meat^\|\|^ | | 26.4 | Sjörs ^(S3)^, Bryngelsson et al. ^(S12)^, Ceije ^(S16)^ |
|  | Bacon | | 9.6 | Sjörs ^(S3)^, Bryngelsson et al. ^(S12)^ |
|  | Sausage dish | | 18.2 | Sjörs ^(S3)^, Bryngelsson et al. ^(S12)^ |
|  | Hamburger (beef) |  | 24.0 | Sjörs ^(S3)^, Bryngelsson et al. ^(S12)^, Flysjö ^(S13)^ |
| Poultry | Chicken | | 3.6 | Sjörs ^(S3)^, Bryngelsson et al. ^(S12)^ |
| Fish | Lean fish | | 4.2 | Sjörs ^(S3)^, Ziegler et al. ^(S17)^, Winther et al. ^(S18)^ |
|  | Fat fish^°^ | | 3.6 | Sjörs ^(S3)^, Ziegler et al. ^(S17)^, Winther et al. ^(S18)^ |
|  | Salty fish | | 1.3 | Sjörs ^(S3)^, Ziegler et al. ^(S17)^, Winther et al. ^(S18)^ |
|  | Smoked fish^∍^ | | 4.4 | Sjörs ^(S3)^, Ziegler et al. ^(S17)^, Winther et al. ^(S18)^ |
| Sugar-containing foods and snacks | Sweets | | 2.8 | Sjörs ^(S3)^, RISE ^(S14)^, Röös ^(S15)^, Nilsson et al. ^(S19)^ |
|  | Sugar, honey, marmalade, jam | | 4.3 | Sjörs ^(S3)^, Bryngelsson et al. ^(S12)^, Röös ^(S15)^ |
|  | Chips, popcorn, salty nuts | | 1.8 | Sjörs ^(S3)^, Bryngelsson et al. ^(S12)^, Nilsson et al. ^(S19)^ |
|  | Cookies and pastries | | 1.2 | Sjörs ^(S3)^, Bryngelsson et al. ^(S12)^, Nilsson et al. ^(S19)^ |
| Drinks | Soda, juice | | 0.6 | Sjörs ^(S3)^, Röös ^(S15)^, Nilsson et al. ^(S19)^ |
|  | Rosehip soup | | 0.9 | Sjörs ^(S3)^, Röös ^(S15)^ |
|  | Coffee | | 0.2 | Sjörs ^(S3)^, Nilsson ^(S20)^ |
|  | Tea | | 0.04 | Sjörs ^(S3)^, Nilsson ^(S20)^, Scarborough et al. ^(S21)^ |
|  | Class I beer (<2,25%) | | 0.9 | Hallström et al. ^(S22)^ |
|  | Class II beer (2,8-3,5%) | | 0.8 | Hallström et al. ^(S22)^ |
|  | Class III (>3,5%) | | 0.9 | Hallström et al. ^(S22)^ |
|  | Wine | | 2.3 | Hallström et al. ^(S22)^ |
|  | Distilled beverage e.g. vodka | | 2.2 | Hallström et al. ^(S22)^ |

^*^Based on Swedish and imported fresh berries (69,5%), Swedish and imported frozen berries (29,5%) and flight imported berries from South America (1%).

^†^Based on domestically produced fruit (10%), imported fruit (89%) and flight imported fruit (1%), excluding citrus fruit and banana.

^‡^Based on tomato (40%) and cucumber (60%). The tomato is representative of Swedish consumption based on a weighting of 14% Swedish-grown tomato (greenhouse 25% fossil), 59% import from Central Europe (greenhouse, 50% fossil, 50% waste heat) and 27% import from southern Europe (50% free-range cultivation, 50 % non-heated greenhouse). The cucumber is representative of Swedish consumption based on a weighting of 45% Swedish-grown cucumber (greenhouse 60% fossil), 22% import from Central Europe and 33% import from southern Europe (non-heated greenhouse).

^§^Based on light (27%) and full fat (73%) crème fraiche and cream.

^¶^Based on liver pate (50%) and salami fat content 33% (50%). Factors for weighting the proportion of meat and vegetables in liver pate and sausage; liver pate 31% meat, 69% vegetable; salami 96% meat, 4% vegetables.

^||^Based on weighting of beef (41%), lamb (5%), pork (49%) and game (5%). Calculated climate impact for beef based on weighted values of 65.6% beef (not dairy animals), 20.6% dairy bull, 13.8% dairy cow.

^°^Based on salmon (65%), herring (24%) and mackerel (11%).

^∍^Based on salmon (85%) and mackerel (15%).

**References**

S1. IPCC, 2007: Climate Change 2007: Synthesis Report. Contribution of Working Groups I, II and III to the Fourth Assessment Report of the Intergovernmental Panel on Climate Change [Core Writing Team, Pachauri, R.K and Reisinger, A. (eds.)]. IPCC, Geneva, Switzerland: IPCC;2007. 104 pp.

S2. IPCC. Climate Change 2014: Synthesis Report. Contribution of Working Groups I, II and III to the Fifth Assessment Report of the Intergovernmental Panel on Climate Change [Core Writing Team, R.K. Pachauri and L.A. Meyer (eds.)] Geneva, Switzerland: IPCC; 2014. 151 pp.

S3. Sjörs C. Näringsintag och utsläpp av växthusgaser från svenska matvanor ur ett epidemiologiskt perspektiv (Nutritional intake and greenhouse gas emissions from Swedish eating habits from an epidemiological perspective) [in Swedish] [licentiate thesis]. Stockholm; E-print AB Stockholm; 2017 [cited 2018-03-02].

S4. The Swedish National Food Administration. Livsmedelsdatabasen (Food Composition Database) [in Swedish] [Internet]. 2018 [updated 2018-02-15; cited 2018-03-02]. Retrieved from: https://www.livsmedelsverket.se/livsmedelsdatabasen

S5. Ahlmén K./ Sigill Kvalitetssystem AB. Maten och miljön. Livscykelanalys av sju livsmedel (The food and the environment. Life cycle analysis of seven food groups) [in Swedish] [Internet]. LRF; 2002.

S6. Quested TE, Parry AD, Easteal S et al. (2011), Food and drink waste from households in the UK. Nutrition Bulletin, 36: 460-467.

S7. Gustavsson J, Cederberg C, Sonesson U et al. The methodology of the FAO study:”Global Food Losses and Food Waste – extent, causes and prevention” – FAO, 2011 [Internet]. SIK 2013. SIK Report 857.

S8. Central Bureau of Statistics. Statistikdatabasen (Statistical database) [in Swedish] [Internet]. 2018 [updated 2018-03-07; cited 2018-03-07]. Retrieved from: http://www.statistikdatabasen.scb.se/pxweb/sv/ssd/?rxid=f45f90b6-7345-4877-ba25-9b43e6c6e299.

S9. The Department of Agriculture. Hur stor andel av livsmedlen som säljs på marknaden är producerade i Sverige? (How much of the food sold on the market is produced in Sweden?) [in Swedish]. Sweden: The Department of Agriculture; 2017.

S10. The Department of Agriculture. Livsmedelskonsumtion och näringsinnehåll – Uppgifter till och med 2016 (Food consumption and nutritional content - Data through 2016) [in Swedish] [Internet]. 2017 [updated 2017-12-08; cited 2018-03-02]. Retrieved from: http://www.jordbruksverket.se/webdav/files/SJV/Amnesomraden/Statistik, fakta/Livsmedel/JO44SM1701/JO44SM1701_tabeller.htm

S11. Ziegler F, Bergman K. Svensk konsumtion av sjömat – en växande mångfald. (Swedish consumption of seafood- a growing diversity) [in Swedish] Gothenburg: Research Institutes of Sweden (RISE); 2017. SP Report 2017:07

S12. Bryngelsson D, Wirsenius S, Hedenus F et al. How can EU climate targets be met? A combined analysis of technological and demand-side changes in food and agriculture. Food Policy. 2016 Feb;59:152-164. .

S13. Flysjö A. Greenhouse gas emissions in milk and dairy product chains: Improving the carbon footprint of dairy products [doctoral thesis]. Aarhus University, Denmark. 2012.

S14. RISE Climate Food Database 1.4. RISE - Research Institutes of Sweden. Gothenburg.

S15. Röös E. Mat-klimat-listan (Food climate list) [in Swedish]. Version 1.1. Uppsala: SLU - 2014. Report 077. ISSN 1654-9406

S16. Cejie J. Klimatpåverkan från vilt kött (Climate impact from game meat) [in Swedish] [Internet]. 2008 [updated 2008-11-05; cited 2018-03-02]. Retrieved from: http://ekotank.blogspot.se/2008/11/klimatpverkan-frn-vilt-ktt.html.

S17. Ziegler F, Winther U, Hognes E et al. The carbon footprint of Norwegian seafood products on the global seafood market. Journal of Industrial Ecology. 2013;17(1):103-16.

S18. Winther U, Ziegler F, Hognes E et al. Carbon footprint and energy use of Norwegian seafood products. SINTEF Fisheries and Aquaculture, Report SFH80 A. 2009;96068.

S19. Nilsson K, Sund V, Florén B. The environmental impact of the consumption of sweets, crisps and soft drinks. Copenhagen: Nordic Council of Ministers; 2011. TemaNord 2011:509. .

S20. Nilsson K. Klimatpåverkan från bryggkaffe och snabbkaffe. (Climate impact of filter coffee and instant coffee) [in Swedish] SIK - The Swedish Institute for Food and Biotechnology, Gothenburg, Sweden. 2010. Report UPX00221.

S21. Scarborough P, Appleby PN, Mizdrak A et al. Dietary greenhouse gas emissions of meat-eaters, fish-eaters, vegetarians and vegans in the UK. Climatic change. 2014;125(2):179-92.

S22. Hallström E, Håkansson N, Åkesson A et al. Climate impact of alcohol consumption in Sweden. J Clean Prod. 2018; 201: 287-294.

| **Example of calculation of weighted average emissions based on Swedish consumption statistics** | **Example of calculation of emissions of composite dish according to recipe from the national food composition database** |
| --- | --- |
| Weighted average GHG emissions for meat, excluding poultry;  Consumption shares; pork 49%, beef 41%, sheep meat (mutton) 5%, and game 5%.  Kg CO_2_e/kg prepared meat; pork 9.6, beef 45.9, mutton 51.7 and game 6.7.  Calculation of weighted average emission; 0.49*9.6 + 0.41*45.9 + 0.05*51.7 + 0.05*6.7 = 26.4 kg CO_2_e/kg prepared meat. | GHG emissions for hamburger, based on beef;  Proportion of ingredients; hamburger meat (beef) 50%, bread 44%, and cheese 6%.  Kg CO_2_e/kg ingredient; hamburger meat (beef) 45.9, bread 0.7, cheese 11.8  Calculation of weighted average emission; 0.5*45.9 + 0.44*0.7 + 0.06*11.8 = 24.0 kg CO_2_e/kg prepared hamburger |
